# Photosynthetic Solutions for Organ Perfusion Based on Microalgae and Cyanobacteria Display Differential *In Vitro* and *In Vivo* Features for Intravascular Oxygenation

**DOI:** 10.1021/acsabm.5c01137

**Published:** 2025-08-07

**Authors:** Daniela Becerra, Valentina Vargas-Torres, Valentina Veloso-Giménez, Daniela Gallardo-Agüero, Miguel Miranda, Valentina Hernández-Pavez, Nicolás González-Quezada, Sebastián San Martín, Mauricio P. Boric, José Tomás Egaña

**Affiliations:** † Institute for Biological and Medical Engineering, 28033 Pontificia Universidad Católica de Chile, Av. Vicuña Mackenna 4860, Santiago 7820436, Chile; ‡ Center of Interdisciplinary Biomedical and Engineering Research for Health (MEDING), School of Medicine, 28068 Universidad de Valparaíso, Angamos 655, Viña del Mar 2540064, Chile

**Keywords:** photosynthetic therapies, organ perfusion, photosynthetic microorganisms, Chlamydomonas reinhardtii, Synechococcus elongatus, biological oxygen carriers, renal preservation, microbial biocompatibility

## Abstract

The delivery of photosynthetic microorganisms has emerged as a strategy for tissue oxygenation, offering a promising approach to treat several hypoxic conditions. Among these, intravascular photosynthesis has been proposed for *ex vivo* organ preservation; however, the most suitable photosynthetic microorganisms and their behavior during intravascular perfusion remain to be fully elucidated. Therefore, this study evaluates key properties of photosynthetic solutions for organ perfusion, based on the microalgae and the cyanobacterium . *In vitro* characterization showed that both microorganisms maintained viability, morphology, and oxygen production capacity in a Ringer’s lactate-based medium for at least 24 h, with both photosynthetic solutions exhibiting rheological properties compatible with organ perfusion. *In vivo* perfusion of rat kidneys demonstrates sustained hemodynamic stability, with showing lower variability in vascular resistance. Histological analysis revealed significant retention of both microorganisms within renal structures, with inducing less tubular damage. Additionally, biocompatibility assays with human endothelial cells and zebrafish larvae showed no significant cytotoxic effects of the photosynthetic solutions. These findings support the feasibility of using photosynthetic microorganisms for intravascular photosynthesis, highlighting as particularly promising due to its lower oxygen consumption in darkness and reduced tissue damage after perfusion. This work provides significant insights toward the development of biologically active perfusion systems for innovative preservation strategies for organ transplantation.

## Graphical abstract

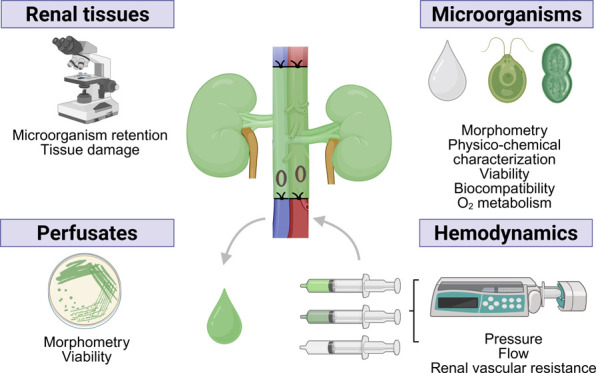

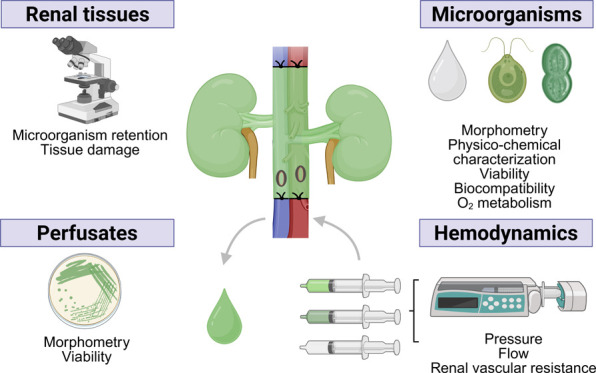

## 1 Introduction

The use of photosynthetic microorganisms for oxygen delivery to tissues has garnered significant attention, with numerous studies confirming the safety and efficacy of this approach. *In vitro* assays have demonstrated biocompatibility with various cell types, such as fibroblasts^1^ and endothelial cells.^2^ *In vivo* studies further support these findings, with successful applications in different models such as zebrafish,^3,4^ mice,^2^ rats,^5^ and even humans.^6,7^ This demonstrated biocompatibility, coupled with the natural capacity of photosynthetic cells to produce and release oxygen in the presence of light, opens novel possibilities for treating hypoxic conditions. Thus, promising results have been achieved in wound healing,^8^ ischemic stroke,^9^ and tumor treatment.^10^

Given the immense biodiversity of photosynthetic microorganisms and the need to match each clinical need with suitable microorganisms, several species have been explored. Among microalgae, is extensively studied for its genetic modifiability and ability to sustain oxygen levels in hypoxic environments.^1,2,12,13^ Similarly, the cyanobacterium has demonstrated significant potential for oxygen delivery in tissue regeneration and cancer therapy.^14^ Moreover, genetically engineered strains have further expanded these possibilities, enabling photosynthetic cells to secrete therapeutic molecules such as growth factors^12,15^ and glycosaminoglycans.^16^ This versatility suggests potential applications beyond tissue oxygenation.

Most research has been focused on implanting photosynthetic cells into hypoxic tissues or scaffolds. However, recent studies propose that circulating photosynthetic cells could serve as erythrocyte substitutes, generating oxygen directly into the bloodstream.^13,17,18^ Proof-of-concept studies using photosynthetic solutions containing microorganisms have demonstrated biocompatibility and sustained oxygen production in zebrafish larvae and rat kidney slices,^18^ holding transformative potential for targeted oxygen delivery to ischemic tissues during surgery or *ex vivo* organ preservation.

Although the concept of vascular photosynthesis is promising, further research must address key scientific and technological challenges before the clinical translation of circulating oxygen-producing cells, or *chlorocytes*.^19^ Among these challenges, understanding the interactions between circulating microorganisms and the vasculature is crucial, particularly regarding biocompatibility, hemodynamics, and vascular clearance. Therefore, this study performed a comprehensive *in vitro* and *in vivo* analysis to evaluate and compare two photosynthetic solutions for vascular photosynthesis, containing well-described model organisms of microalgae and cyanobacteria.

## 2 Materials and Methods

### 2.1 Strains and Growth Conditions

Cell-wall deficient microalgae (cw15–30-derived) UVM4 ^20^ were kindly provided by Profesor Joerg Nickelsen (LMU) and grown in Tris-Acetate-Phosphate (TAP) medium^21^ as previously described.^13^ Microalgal density was determined by counting in a Neubauer chamber.

 cyanobacteria (strain UTEX 2973) were purchased at the UTEX Algae Collection, University of Texas. Cultures were grown at 37 °C under continuous white LED light exposure (90 μE/m^2^s) on either solid Blue Green 11 (BG11) medium (1.5% w/v agar) or liquid BG11 medium^22^ in T25 or T75 cell culture flasks with a ventilated cap. Flasks were placed upright on an orbital shaker (130 rpm). In all cases, solid and liquid BG11 media were supplemented with 20 mM HEPES (pH 8, AppliChem Panreac). The cyanobacterial growth was measured by optical density at 750 nm (OD_750_) and was calculated via linear regression with the following formula (obtained from experimental data)

$$\mathrm{million}\;\mathrm{of}\;\mathrm{cells}/\mathrm{mL}=\left({\mathrm{OD}}_{750}-0.04249\right)/0.0007628$$

### 2.2 Generation of Photosynthetic Perfusable Solution for Organ Perfusion

Ringer’s lactate solution (Fresenius Kabi) was supplemented with 0.5% (w/v) mannitol (AppliChem Panreac) and 5% (w/v) dextran-70 (AK Scientific Inc.) to obtain RLMD solution. Photosynthetic solutions for organ perfusion (PSOP) were generated by resuspending microorganisms in RLMD in amounts required to sustain similar oxygen production. Liquid cultures of or in the exponential growth phase were centrifuged at 2000 rpm for 5 min or 4500 rpm for 10 min, respectively. Pelleted microalgae and cyanobacteria were resuspended in RLMD at 1 × 10^8^ /mL and 3 × 10^9^ /mL and filtered with a 40 μm cell strainer.

### 2.3 Viability Assays

#### 2.3.1 Growth in Agar Plates

After 0 or 24 h of incubation in RLMD, 10 μL of cyanobacteria-based PSOP were spread evenly on a 35 mm BG11 agar plate. Microalgae-based PSOP was diluted 40 times, and 12 μL was inoculated by streaking on a 35 mm TAP agar plate. The viability of microorganisms was determined by examining growth after 7 days of inoculation under the respective culture conditions described above.

To assess microalgae and cyanobacteria viability before or after perfusion in rat kidney experiments, 10 μL of each input or perfusate sample was seeded in TAP or BG11 plate, as appropriate. After 7 days of incubation, plates were imaged, and the covered area was determined using Fiji Software v2.0.9.^23^

#### 2.3.2 FDA Staining

The viability of microalgae in RLMD solution was determined at 0 and 24 h of incubation by flow cytometry (BD FACSCanto II Analyzer, Becton Dickinson) using fluorescein diacetate (FDA, F1303, Life Technologies) as previously described.^13^ Data were analyzed using FlowJo Software v10 (Becton Dickinson) by gating singlets, then selecting chlorophyll-positive cells, and finally determining the percentage of FDA-positive cells within the chlorophyll-positive population.

#### 2.3.3 MTT Assay

MTT assay was performed to determine the viability of cyanobacteria. After 0 or 24 h of incubation in RLMD, 90 μL of cyanobacteria-based PSOP and 10 μL of MTT (5 mg/mL; Invitrogen) were incubated for 1 h at 37 °C in the dark in a 96-well plate. After incubation, 100 μL of DMSO (AppliChem Panreac) was added to each well, and the plate was incubated for 10 min at room temperature (RT) on a rocker shaker. After that, 50 μL of the content of each well was diluted using 150 μL DMSO, and absorbance was measured at 570 nm. Cyanobacteria-based PSOP density was determined by optical density at OD_750_. Data was normalized and expressed as absorbance at 570 nm per 10^9^ cells.

### 2.4 Cell Morphology Evaluation

Microalgae and cyanobacteria morphology in both PSOP was evaluated by optical microscopy at 0 and 24 h of incubation in RLMD. For imaging, 5 μL of each PSOP were loaded onto a glass slide, and phase contrast images were acquired using optical microscopy equipped with a phase Stop Ph1/0.4 (ZEISS Primovert) using a 40× objective and a standard digital camera (MS60, Mshot). Feret’s diameter and MinFeret quantifications of individual cells were performed with Fiji Software v2.0.9.^23^ For analysis, a threshold was applied to remove the phase halo. Individual particles were then defined by their area and circularity: microalgae were defined as particles with an area of 11.00–80.00 μm^2^ and a circularity of 0.65–1.00, while cyanobacteria were defined as particles with an area of 1.00–18.00 μm^2^ and a circularity of 0.00–0.80.

To analyze microalgae and cyanobacteria morphology in both PSOP before and after perfusion in rat kidney experiments, 5 μL of each sample was loaded onto a glass slide, and fluorescence images were acquired with epifluorescence microscopy (Leica, DM500) and a digital camera (Axiocam, 208 color). Images were analyzed using Fiji Software v2.0.9 ^23^ by determining the number, Feret’s diameter and MinFeret of the particles. Bright-field images were acquired using an optical microscope (ZEISS Primovert) with a 40× objective and a standard digital camera (MS60, Mshot).

### 2.5 Oxygen Consumption and Production Measurements

After 0 and 24 h of incubation in RLMD, microalgae and cyanobacteria oxygen production were measured using an Oxygraph System (Hansatech Instruments) at 28 or 37 °C, respectively. Samples (1 mL) were introduced into the electrode chamber and subjected to 5 min of darkness, followed by 5 min of simultaneous blue (455 nm, 248 μE/m^2^s) and red (630 nm, 317 μE/m^2^s) illumination. The oxygen production rate in the illuminated phase was calculated as the slope of the oxygen concentration curves over time using a Python script.

### 2.6 Osmolality and Viscosity Assessment

After 0 and 24 h of incubation in RLMD, the osmolality of both PSOP was measured at RT using a cryoscopic osmometer (Osmomat 030, Gonotec). The osmolality of RLMD was recorded as a control. For the viscosity assessment, an HR-2 rheometer (Discovery) was employed. These measurements were conducted at RT and atmospheric pressure, using the 25 mm parallel plate geometry with a gap of 300 μm and utilizing a sample volume of 150 μL per measurement. The viscosity of the solution was recorded at increasing shear rates of 15–1000 (1/s).

### 2.7 Determination of the Content of Photosynthetic Cells in PSOP Volume

The volume percentage of microorganism in the total PSOP volume (equivalent to hematocrit for blood samples) was determined in nonheparinized hematocrit glass capillaries. Samples were prepared at concentrations 10 times larger than those used in PSOP preparation, meaning concentrations of 1 × 10^9^ /mL and 3 × 10^10^ /mL in RLMD. Tubes were filled by capillarity, sealed at one end using sealant clay, and centrifuged at 12,800 rpm for 10 min at RT using a centrifuge equipped with a hematocrit rotor (Pico 17 microcentrifuge, Heraeus, Thermo Scientific). The proportion of photosynthetic cells in the PSOP volume was calculated by dividing the length of the packed microorganism layer by the total length of the PSOP solution, which includes both microorganisms and supernatant, and then dividing the result by 10. Each sample was assessed in duplicate, and the result represents the average of the two measurements.

### 2.8 HUVEC Culture, Biocompatibility Assay, and Interaction Experiment

Human umbilical vein endothelial cells (HUVECs) were kindly provided by Dr. Claudia Saez and maintained under standard cell culture conditions (37 °C, 5% CO_2_) in human endothelial basal media (EBM-2) supplemented with EGM-2 single quotes supplements and 2% fetal bovine serum (Lonza). In all experimental settings, cells with 3 to 6 passages were used.

For the biocompatibility assay, HUVECs were seeded on 96-well plates (5000 cells/well) and cultured until they reached confluency. Then, or cells were added in a ratio of 10 microalgae or 300 cyanobacteria (in EGM-2 medium without antibiotic) per endothelial cell (i.e., maintaining the microorganisms’ concentration ratio used in each PSOP solution), and then were cocultured for 24 h. Supernatants were collected, and 10 μL were seeded onto BG11 or TAP agar plates to assess the viability of photosynthetic microorganisms. Thereafter, cells were gently washed with EGM-2 medium. For MTT assay, cells were incubated with 90 μL of EGM-2 and 10 μL of MTT (5 mg/mL; Invitrogen) for 1 h in the dark under standard cell culture conditions (37 °C, 5% CO_2_). Then, 100 μL of DMSO were added to each well, and the plate was incubated for 10 min at RT on a rocker shaker. Absorbance was measured at 570 nm in a BioTek Epoch 2 Reader (Agilent Technologies).

For the interaction protocol, HUVECs were seeded on 24-well plates (50,000 cells/well) containing a cover glass treated with poly-l-Lysine (P4707–50 ML, Sigma-Aldrich) and cultured until they reached confluency. Then, or cells were added in a ratio of 10 microalgae or 300 cyanobacteria (in RLMD) per endothelial cell and were cocultured for 1 h. After that, five consecutive washing steps with RLMD were made by pipetting softly. Phase-contrast bright-field images were acquired with a standard microscope (DM500, Leica) equipped with phase Stop Ph1/0.4 and a digital camera (Mshot M60). Photosynthetic microorganisms were quantified using Fiji Software v2.0.9.^23^ For actin quantification, phalloidin staining was employed on unwashed cocultures, exposing the glass coverslips to 1/100 and 1/5000 concentrations of green phalloidin (488 nm) and Hoechst stain, respectively, in 0.1% Triton X-100 in phosphate-buffered saline (PBS) for 20 min. Fluorescence images of the coculture were obtained with a Cytation 5 cell imaging multimode reader and microscope equipped with Gen5 software (Agilent Technologies Inc.). Images were analyzed with the OrientationJ plugin and particle quantification method in Fiji software v2.0.9.^23^

### 2.9 Biocompatibility Assay with Zebrafish

Five zebrafish larvae (, TAB5 strain, 5 dpf) were incubated for 24 h at 28 °C in a 24-well plate with 1 mL of E3 medium (control), RLMD, microalgae-based PSOP, or cyanobacteria-based PSOP under the optimal conditions for larvae as previously described.^18^ Subsequently, survival rates were assessed by determining the percentage of larvae exhibiting heartbeats. For morphological examination, larvae were rendered unconscious by immersion in 4.2% (w/v) tricaine (Sigma-Aldrich), immobilized in 1.5% agarose, and imaged using a stereoscope (Leica S6D) and a digital camera (Mshot M60).

### 2.10 Metabolic Coupling Assay

For metabolic coupling experiments, 20 zebrafish larvae (5 dpf, TAB5 strain) in 1 mL RLMD were placed in the electrode chamber of an Oxygraph System. Oxygen evolution was recorded as previously described^13^ with modifications: Briefly, the samples were incubated for 5 min in darkness, 5 min under blue (455 nm, 248 μE/m^2^s) and red (630 nm, 317 μE/m^2^s) light. Then, 100 μL of RLMD was replaced with either microalgae-based PSOP (1 × 10^9^ /mL) or cyanobacteria-based PSOP (3 × 10^10^ /mL). Oxygen evolution was monitored for 5 min under the same lighting, followed by 5 min in darkness. Experiments were performed at 28 °C for microalgae and 37 °C for cyanobacteria. Oxygen production rate was calculated from the slopes of oxygen concentration curves over time using a Python script.

### 2.11 Surgery Settings for *In Vivo* Kidney Perfusion

Male Sprague–Dawley rats weighing 250–350 g were obtained from the Bioterio Central (CIBEM) animal facility at the Pontificia Universidad Católica in Santiago, Chile. All animal procedures were conducted following a protocol approved by the Ethics Committee of Pontificia Universidad Católica de Chile (protocol N°220808006). Animals were anesthetized intraperitoneally using a combination of ketamine (90 mg/kg) and xylazine (10 mg/kg). Surgical procedure was conducted as described before.^13^ Briefly, the renal vasculature was isolated by ligating the mesenteric artery, as well as the aorta and vena cava, at the infrarenal and suprarenal positions. A cannula was retrogradely inserted into the infrarenal aorta to enable perfusion, and an incision was made in the vena cava to allow the perfusate to exit.

A clinical syringe pump (PG907, Progetti) was used to drive the perfusate through the cannulated aorta, enabling regional perfusion of the renal area. As a flushing step, approximately 40 mL of RLMD solution was perfused through both kidneys over about 10 min to remove blood. Following this, a clinical syringe pump (B. Braun AG) was used to infuse the solutions containing microorganisms through the cannulated aorta. Approximately 35 mL of PSOP solution (either microalgae- or cyanobacteria-based) was perfused through both kidneys over 10 min. After perfusion, the left kidney was clamped and removed with its vasculature filled with PSOP, while the perfusion flow to the right kidney was reduced by half to initiate the rinsing protocol aimed at removing the microorganisms. This rinsing step was performed using the first syringe pump (B. Braun AG), which perfused approximately 20 mL of RLMD solution over 10 min. After the rinsing step, the right kidney was clamped and removed. Then, animals were euthanized with a lethal intracardiac dose of combined ketamine (300 mg/kg) and xylazine (30 mg/kg).

Perfusate samples were collected at 9, 15, 21, 25, and 30 min of perfusion. A control group was conducted, in which RLMD was continuously perfused through steps 1 and 2 in both kidneys and in the remnant right kidney during step 3.

Arterial pressure was monitored with a sensor (P23XL-1, Becton Dickinson) connected to the cannula and the syringe pumps with a T-junction. The pressure was continuously recorded using a data recording module (DI-158U, DataQ Instruments) and WinDaq/XL software (DataQ Instruments), adjusting the input flow to maintain arterial pressure below 120 mmHg.

### 2.12 Histology and Tissue Imaging

#### 2.12.1 Sample Preparation

Whole fresh kidneys were photographed in a stereoscope (Leica S6D) using a conventional digital camera (MS60, Mshot). Then, kidneys were split into two transverse halves using a scalpel. The bottom half was immediately fixed in 4% paraformaldehyde (PFA) at 4 °C for microscopic distribution and vascular integrity analyses. Meanwhile, the upper half of the excised kidneys was promptly sectioned with a scalpel to obtain transverse slices of about 2 mm. From center to pole, one slice was PFA-fixed (24 h, 4 °C) followed by dehydration in 30% sucrose (24 h, 4 °C), while the remaining slices were immediately frozen at −80 °C for further analysis.

#### 2.12.2 Distribution of Microorganisms in the Tissue

Macroscopic distribution was evaluated in the PFA-fixed and sucrose-dehydrated transverse slices obtained from the upper half of each kidney using a stereoscope (Leica S6D) with a conventional digital camera (MS60, Mshot).

The PFA-fixed lower half of each kidney was cut from the center toward the pole into two sections. To determine the microscopic distribution of microorganisms, one of these sections was dehydrated with 30% sucrose, and 10 μm tissue cryosections were prepared for bright-field microscopy using a standard light microscope (CX21, Olympus) and a digital camera (4K Sony Ultra HD VC.3040). The other section was used for histological analysis of tissue integrity, as will be described below.

#### 2.12.3 Histological Assessment of Tissue Integrity

PFA-fixed tissues were embedded in paraffin, yielding 3 μm tissue sections. Samples were then mounted in double poly-l-Lysine-treated microscope slides (Poly-l-Lysine 0.1% w/v in H_2_O, Sigma-Aldrich) and stained with hematoxylin-eosin (H&E) following standard methods. The sections were examined using a standard light microscope (CX21, Olympus) and a digital camera (4K Sony Ultra HD VC.3040). Tissue damage was assessed using the endothelial, glomerular, and tubular (EGT) scoring system, an adapted version of the EGTI scoring system originally described in previous studies,^24^ which rates endothelial, tubular, and glomerular damage from 0 (no damage) to 9 (severe damage).^13^ Samples were examined using standard brightfield microscopy (CX21, Olympus) and a digital camera (4K Sony Ultra HD VC.3040).

#### 2.12.4 Fluorescence Microscopy and Scan Tile of Kidney Slices

The previously described PFA-fixed and dehydrated transverse slices (2 mm) used for macroscopic imaging were mounted in plastic cryomolds, embedded in O.C.T. (Tissue-Tek, Sakura), and cryosectioned at 60 μm. The sections were then transferred to multiwell plates containing PBS. Afterward, the slices were stained with Hoechst (1/5000) overnight at RT in an orbital shaker (60 rpm). Finally, the slices were transferred to positively charged slides (DW3000, Prolab), mounted with Fluoromount-G (00–4958–02, ThermoFisher), and observed under Cytation 5 cell imaging multimode reader and microscope (Agilent Technologies) in z-stacking and mosaic imaging modalities, employing DAPI (excitation 377/50, emission 447/60), GFP (excitation 469/35, emission 525/39), and Cy5 (excitation 628/40, emission 685/40) filters to visualize the nuclei staining (blue), and tissue (green) and photosynthetic microorganisms (red) autofluorescence.

Scan tile setting was utilized to capture complete kidney slice images. Mosaic imaging was generated using a magnification of 4× (13 steps, z-stacking step size: 5 μm). Postprocessing analysis, including automatic stitching and z-projection, was conducted using Gen5 Image Prime software (v3.16, Agilent). Quantification analyses were performed using Fiji Software v2.14.^23^ Briefly, the stitched mosaic images were analyzed manually, demarcating the cortex and medulla and quantifying red signals, covering both separated anatomical areas. Then, selected 4× images (from consistent anatomical areas among kidneys) were analyzed to determine the area covered by the red signal in and out of the glomeruli in the cortical region. Representative 10× images were also acquired in a Cytation 5 cell imaging multimode reader and microscope (Agilent Technologies) in z-stacking modality (60 steps, z-stacking step size: 1 μm) and then processed in ImageJ software^23^ using PSF Generator and DeconvolutionLab2 plugins (Richardson-Lucy algorithm with 10 iterations), followed by z-projection.

### 2.13 Statistical Analysis

Data represents at least three independent experiments with three or more biological replicates (N). Statistical analyses were executed using GraphPad Prism 8.3 software (GraphPad Software). Unless explicitly mentioned, error bars on graphs represent the standard deviation (SD) and each dot represents one biological replicate. The statistical assessment involved determining data normality via the Shapiro-Wilk test or Kolmogorov–Smirnov test as appropriate, followed by *t* tests, Dunn′s nonparametric test, One-way or Two-way ANOVA followed by Tukey’s multiple comparison tests or Sidak’s multiple comparisons test, as applicable. Significance between groups was established at *p* < 0.05. Further specifics are provided within each figure’s legend.

## 3 Results

### 3.1 PSOP Characterization

Since the optimal growth media for and differ from standard mammalian solutions, the effect of RLMD on these microorganisms was evaluated. Cells were resuspended in RLMD and showed no apparent morphological changes after 24 h (Figure 1A). Quantitative analysis revealed the formation of cyanobacterial clusters at 0 h, which persisted up to 24 h and represented approximately 25% of the total analyzed particles. In contrast, no cluster formation was observed for microalgae (data not shown). The analysis also demonstrated that the length (Feret’s diameter) and width (MinFeret) of microorganisms were maintained over time (Figure 1B). When comparing the length of microalgae and individual cyanobacteria, a significant difference was observed at 0 h (5.73 ± 0.30 and 5.17 ± 0.36 μm, respectively). After 24 h of incubation in RLMD, a slight but not significant difference was observed (5.43 ± 0.17 and 5.13 ± 0.24 μm, respectively). In contrast, the width of the cyanobacteria was significantly smaller than that of the microalgae, being approximately 2.5 times lower at both time points (0 h: 5.10 ± 0.34 and 2.06 ± 0.05 μm; 24 h: 5.00 ± 0.16 and 2.03 ± 0.05 μm for microalgae and cyanobacteria, respectively). Then, the growth capacity of the microorganism was evaluated by agar plating, with plates imaged after 7 days. As a result, both microorganisms were able to grow after incubation in RLMD (Figure 1C). Additionally, no significant differences in viability were observed after 0 or 24 h of incubation in RLMD, as assessed by FDA staining for microalgae and MTT assays for cyanobacteria (Figure 1D).

**Figure 1:**
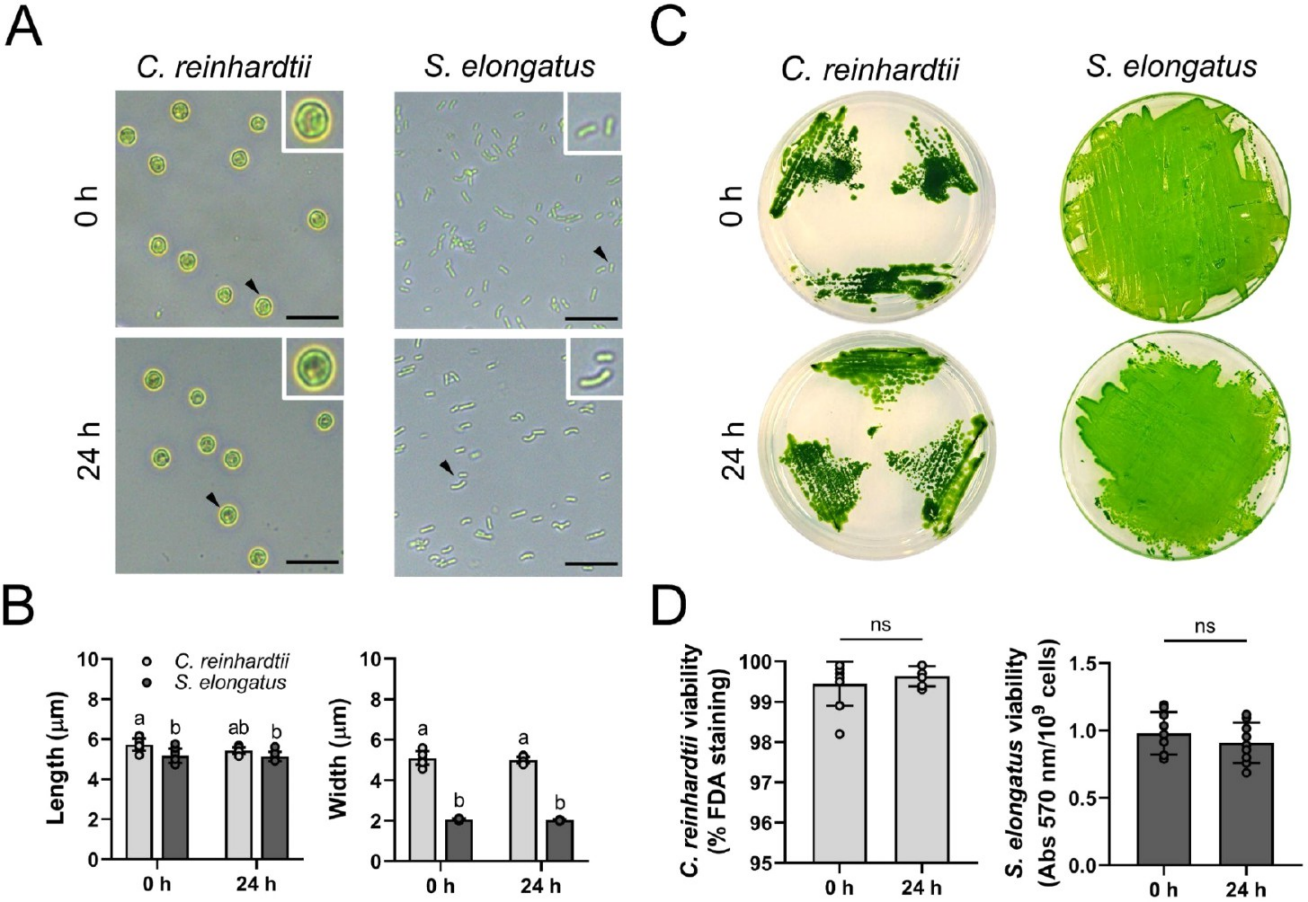
Effect of RLMD on photosynthetic microorganisms. The microalgae and the cyanobacteria were incubated in RLMD for 0 or 24 h at 28 and 37 °C, respectively. (A) Brightfield images showing the morphology of the microorganisms. A digital zoom (2×) is shown in each image. Black arrowheads show the magnified microorganism. (B) Quantification of particle length and width. (C) Proliferation capacity of both microorganisms in agar plates after 7 days of culture. (D) Viability of and was quantified using FDA staining and MTT absorbance at 570 nm, respectively. Statistical analysis was performed using Two-way ANOVA followed by Tukey’s multiple comparison tests in (B) (*N* = 9), Shapiro-Wilk test and Mann–Whitney test in (D, left) (*N* = 9), and Shapiro-Wilk test and Student’s *t* test in (D, right) (*N* = 9). Data are presented as mean ± SD. In (B), statistical significance between groups is indicated by different letters (*p* < 0.05). In (D), “ns” denotes nonsignificant differences. Scale bars represent 20 μm in (A).

After confirming that the morphology, proliferative capacity, and viability of and remained unaffected for at least 24 h in RLMD, key functional features of both PSOP were evaluated and compared. In the darkness, the cyanobacteria-based PSOP exhibited lower oxygen consumption rates at both 0 and 24 h of incubation compared to the microalgae-based PSOP (Figure 2A, left). In the presence of light, the microalgae-based PSOP showed a higher oxygen production rate at 0 h, but no significant differences were observed between the both PSOP after 24 h (Figure 2A, right).

**Figure 2:**
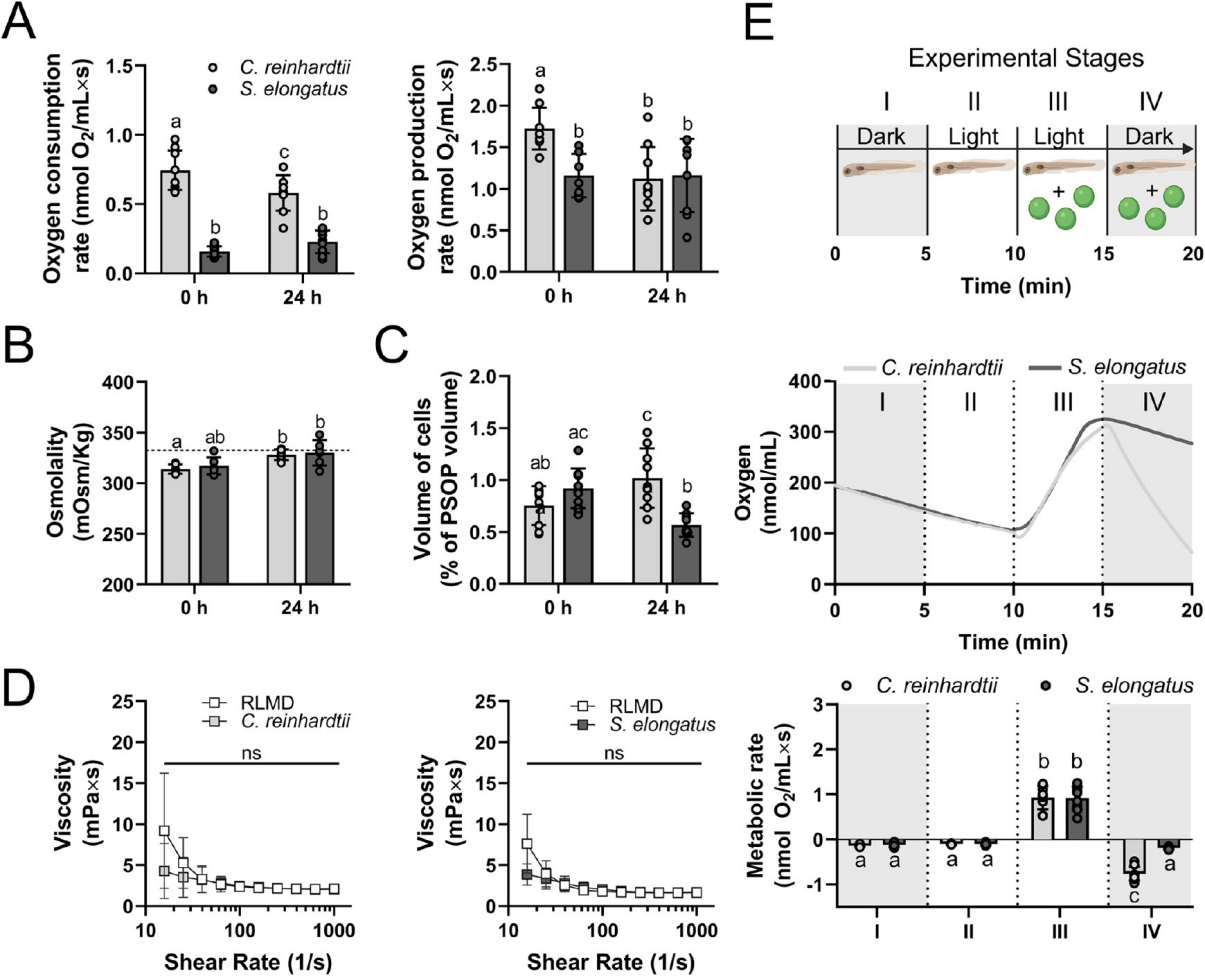
Physicochemical and functional characterization of photosynthetic solutions for organ perfusion. Solutions containing either the microalgae or cyanobacteria were characterized in terms of (A) oxygen consumption in darkness and production under light, (B) osmolality, (C) cell volume relative to the total volume of the solution at 0 and 24 h of incubation in RLMD, and (D) viscosity of freshly prepared PSOP. Additionally, the functional oxygenation capacity of both PSOP was evaluated in zebrafish larvae. (E, upper) shows the experimental setup, (E, middle) displays a representative example of the oxygen evolution curve, and (E, lower) presents the metabolic rates in each stage. In (A) and (D), measurements were performed at 28 °C for *C*. *reinhardtii* and 37 °C for . Statistical analysis was performed using Two-way ANOVA followed by Tukey’s multiple comparison test in (A–C) and (E) (*N* = 9) and Two-way ANOVA followed by Sidak’s multiple comparisons test in (D) (*N* ≥ 9). Data are presented as mean ± SD. Statistical significance is indicated by different letters (*p* < 0.05) in (A–C) and (E), and “ns” denotes nonsignificant differences. The dotted line in (B) represents the viscosity of the empty RLMD solution. Schematic in (E, upper) was created using Biorender (www.biorender.com).

Regarding physical properties, osmolality was quantified, and the microalgae-based PSOP showed a slight but significant increase from 0 to 24 h. Moreover, both PSOP maintained physiological levels (Figure 2B). Additionally, the total volume occupied by the photosynthetic cells was evaluated, showing that approximately 1% of the volume corresponded to cells at both 0 and 24 h post-PSOP preparation, exhibiting a small increase in cell content in the microalgae-based solution and a small decrease in the cell content in the cyanobacteria-based solution at 24 h (Figure 2C). Finally, viscosity measurements revealed no differences of either PSOP solution as compared with the RLMD vehicle solution (Figure 2D), all of them showing a tendency for shear-thinning rheological behavior with viscosity increasing at low shear rates.

Next, *in vivo* tests were performed to evaluate the capacity of both PSOP to meet the metabolic oxygen requirements of an active biological system. As shown in the schematic (Figure 2E, upper panel), zebrafish larvae were introduced into the oxygraph chamber, and oxygen concentration was measured for 5 min in darkness (stage I), followed by 5 min of light (stage II). Then, microalgae or cyanobacteria were incorporated in the RLMD, and measurements were taken for 5 min in light (stage III), followed by 5 min in darkness (stage IV). Here, in the absence of microorganisms (stages I and II), oxygen evolution showed a negative slope (Figure 2E, middle panel), indicating that the oxygen consumption of the larvae is independent of light. Once microalgae or cyanobacteria were incorporated, the oxygen evolution in the presence of light (stage III) showed a positive slope, indicating that the oxygen production of the solutions exceeded the consumption rate of the larvae. However, when the light was turned off (stage IV), a negative slope was observed again for both PSOP, but it was steeper for the microalgae-based PSOP. Finally, metabolic rates were calculated (Figure 2E, lower panel), and oxygen production rates in stages I, II, and III did not show statistical differences between the microorganisms. In contrast, at stage IV, a significant decrease in the slope was determined for the cyanobacteria-based PSOP.

To evaluate biocompatibility, HUVECs were cocultured with photosynthetic microorganisms, and their viability was confirmed through a metabolic assay (Figure 3A) and growth capacity (Figure 3B), respectively. Additionally, zebrafish larvae were incubated in both PSOP, and the effect on larval morphology and survival was assessed. No differences in morphology were observed (Figure 3C); however, a slight but significant decrease in larval survival was detected in the -based PSOP (Figure 3D).

**Figure 3:**
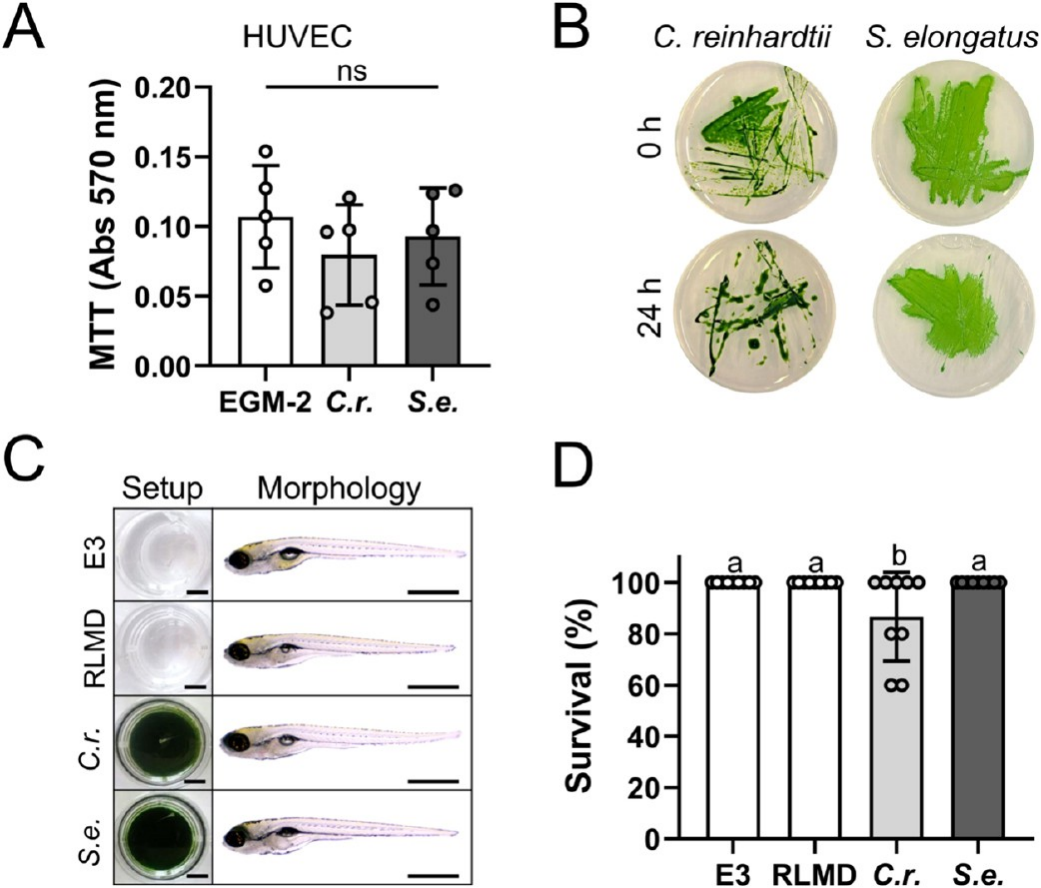
Biocompatibility of photosynthetic microorganisms *in vitro* and *in vivo*. (A) Viability of HUVEC after 24 h of coculture at 37 °C with endothelial basal medium plus supplements (EGM-2), , or quantified by MTT assay. (B) Proliferation capacity in agar plate of or after 24 h of coculture at 37 °C with HUVEC. (C) Morphology of zebrafish larvae (6 dpf) after 24 h of incubation at 28 °C in standard medium (E3), RLMD without microorganisms, or containing or . (D) Survival rate of the zebrafish larvae after incubation. Statistical analysis was performed using the Shapiro-Wilk test, one-way ANOVA, and Tukey’s multiple comparison test in (A) (*N* = 5) and Kolmogorov–Smirnov test, Kruskal–Wallis test, and Dunn’s multiple comparisons test in (D) (*N* = 9). Data are presented as mean ± SD. Statistical significance is indicated by different letters (*p* < 0.05). In (A), (C) and (D) *C*.*r*. represents the microalgae , and *S.e*. represents the cyanobacteria . The scale bar represents 5 mm and 500 μm, and (C, left) and (C, right), respectively.

### 3.2 *In Situ* Perfusion of Rat Kidneys with PSOP

Then, rat kidneys were perfused *in situ* with these solutions to evaluate and compare hemodynamics and tissue and perfusate outcomes. The experimental setting included three steps: First, a flushing step to remove blood from the vasculature, then a perfusion step with one of the PSOP, and a final rinsing step to eventually remove the microorganisms from the renal vasculature (Figure 4A and Video 1, Supporting Information). Throughout these steps, the pressure and perfusion flow were recorded, and the renal vascular resistance (RVR) was calculated. There were no differences in these variables among the experimental groups, with all curves overlapping (Figures 4B, and S1A,B). A tendency toward an increase in RVR was observed for both PSOP during the perfusion step, but it was not statistically significant (Figure 4B). In the cyanobacteria-based PSOP group, RVR returned to baseline during the rinsing step, while in the microalgae group, it remained elevated throughout the rinsing step; however, these differences were not statistically significant. Bar graphs confirm the similarities of all the solutions studied, showing a higher variability for the microalgae-perfused group, especially at the rinsing step (Figure 4C).

**Figure 4:**
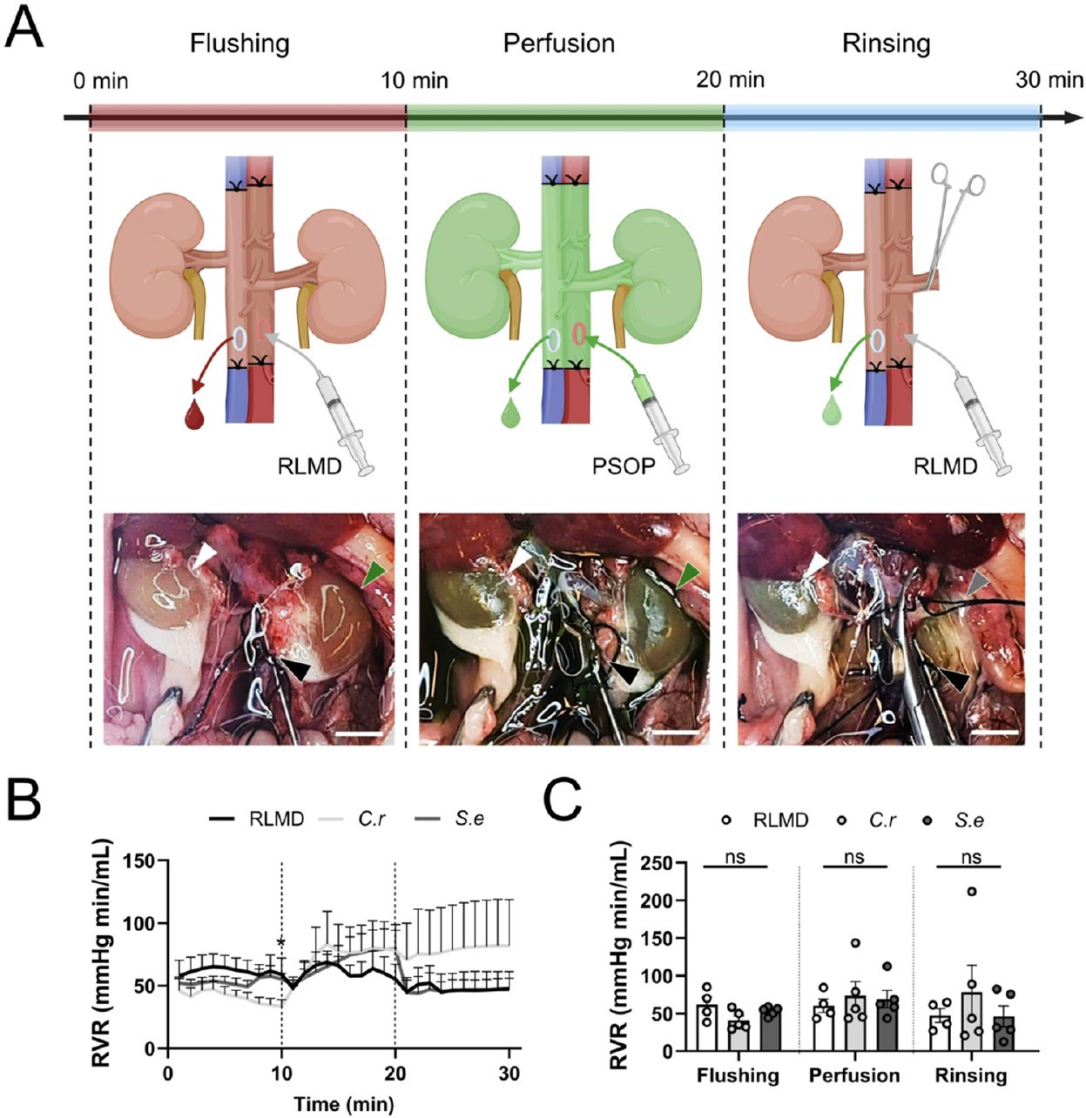
Hemodynamics of photosynthetic solutions for organ perfusion. (A) Schematic representation of the experimental setup (upper panel) along with representative pictures of each step (lower panel). A black arrowhead indicates the site of cannulation in the aorta. The rinsed and perfused kidneys are shown by white and green arrowheads, respectively. A gray arrowhead indicates the location where the perfused kidney was placed prior to being removed. (B) Time-course of renal vascular resistance (RVR) during the perfusion process. (C) The mean of RVR at each stage is represented in bar graphs. Data are presented as mean ± SEM in (B, C). Statistical analysis was performed using Two-way repeated measures ANOVA, followed by Tukey’s multiple comparison test in (B, C) (*N* ≥ 4). Significant differences were observed only between groups *C*.*r*. and *S*.*e*. at time 10 min in (B) (*p* < 0.05), and no significant differences (ns) were observed in (C). In (B, C), *C*.*r*. represents the microalgae and *S*.*e*. represents the cyanobacteria . Scale bar represents 1 cm in (A). Schematic in (A) was created using Biorender (www.biorender.com).

In parallel, the effect of the perfusion procedure on microorganisms was also investigated. For this, perfusate samples were collected from the vein outflow at five different time points: one sample at the end of the flushing step, another during the perfusion step and three samples at the rinsing step (Figure 5A). As expected, the brightfield microscopy (Figure 5B) showed the presence of abundant microorganisms in both PSOP before perfusion and erythrocytes in perfusate 1. In perfusate samples 2 to 5, a mixture of both cell types was observed, with their overall morphology and color remaining intact throughout the process. Quantitative analysis of the images (Figure 5C) showed that the density number of both microorganisms, and , exhibited a strong tendency to decline over the rinsing step, reaching statistical significance at perfusate 5 as compared to the previous steps for both microorganism groups. In terms of size and shape (length and width), the results showed that microorganisms recovered in the outflow were comparable to the respective control PSOP inflow before perfusion. Importantly, clusters of cyanobacteria (particles with an area >18.00 μm^2^) were observed in the PSOP and perfusates 2 and 3.

**Figure 5:**
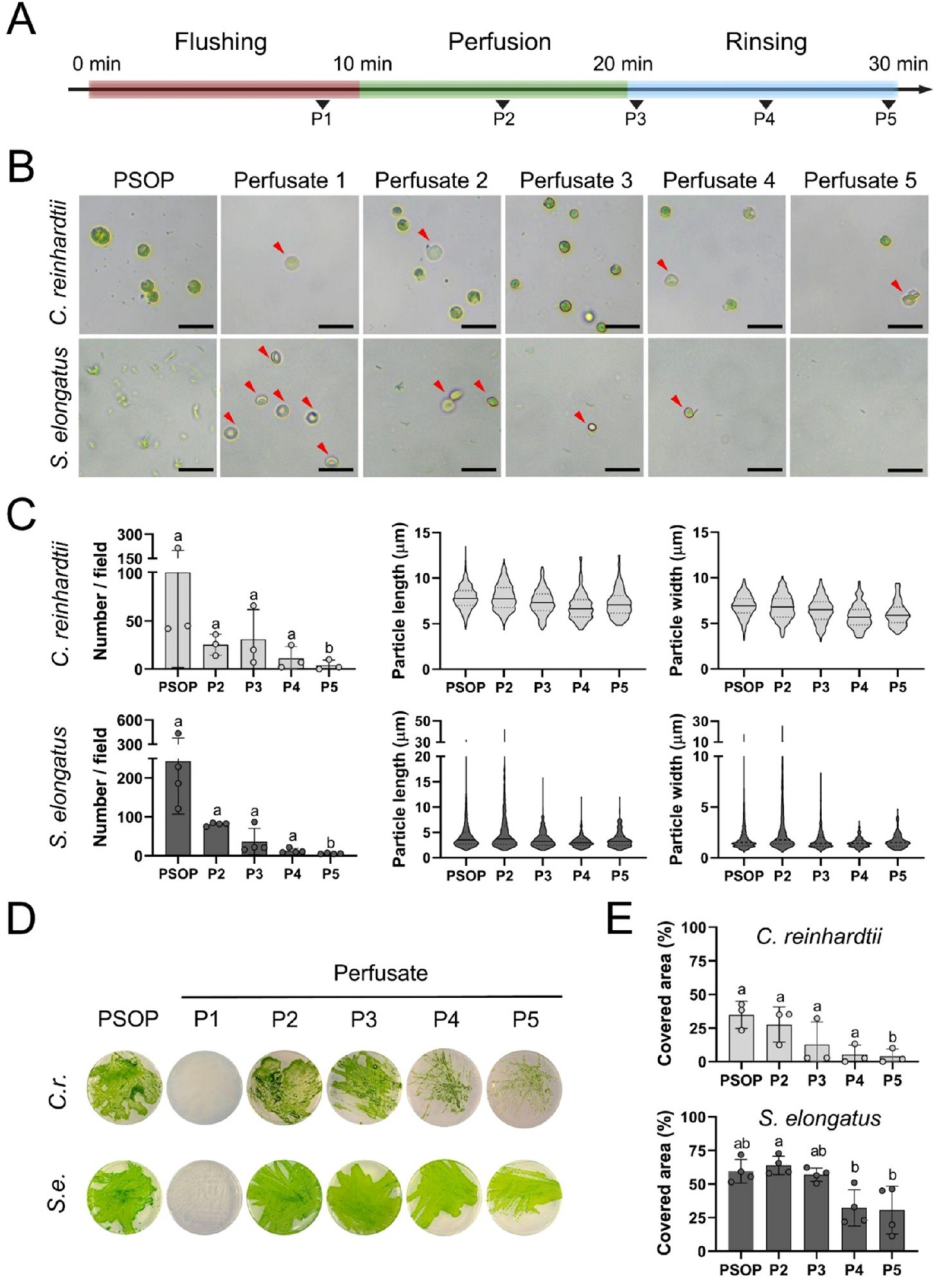
Characterization of the perfusates collected from kidneys. (A) Schematic representation of the experimental setup and sampling times of perfusates, indicated by black arrowheads. (B) Representative bright field images of the different samples. Red arrowheads indicate erythrocytes. (C) Quantification of particle number, length, and width per field. In violin plots, continuous lines show the median, and dotted lines show the interquartile range. (D) Representative images of agar plates seeded with the perfusate samples. (E) Quantification of the covered area on the agar plates. Statistical analysis was performed using Shapiro-Wilk test, Friedman test, and Dunn’s multiple comparisons test in (C, top and bottom, bar graphs) and (E, top) (*N* ≥ 3) and Shapiro-Wilks test, repeated measures ANOVA and Tukey’s multiple comparisons test in (E, bottom) (*N* = 4). Data are presented as mean ± SD. Statistical significance is indicated by different letters (*p* < 0.05). In (D), *C.r*. represents the microalgae and *S.e*. represents the cyanobacteria . Scale bars represent 20 μm in (B). Schematic in (A) was created using Biorender (www.biorender.com).

Finally, the recovered microorganisms were seeded on agar plates, and their viability and proliferation capacity were evaluated by direct visualization (Figure 5D) and further quantified by measuring the covered growth surface on the plates (Figure 5E), showing a significant reduction in surface coverage for perfusate 4 in , and in perfusate 4 and 5 in .

### 3.3 Distribution and Retention of Photosynthetic Microorganisms in the Kidney Vasculature

To assess the distribution of the photosynthetic microorganisms throughout the kidneys and evaluate the effect of the rinsing step, whole organs were imaged and then sliced to visualize the cortical and medullar structures (Figure 6A). Overall, both perfused and rinsed organs exhibited the characteristic green coloration of their respective PSOP, with a marked presence in the vascular structures of the glomeruli and medulla. Unexpectedly, no apparent differences were observed between the perfused and rinsed groups. These results were further confirmed in tissue cryosections, where cyanobacteria displayed a more diffuse and heterogeneous distribution within the vascular networks compared to microalgae-perfused kidneys (Figure 6B). Noteworthy, there was no indication of the presence of either microorganism outside vascular structures.

**Figure 6:**
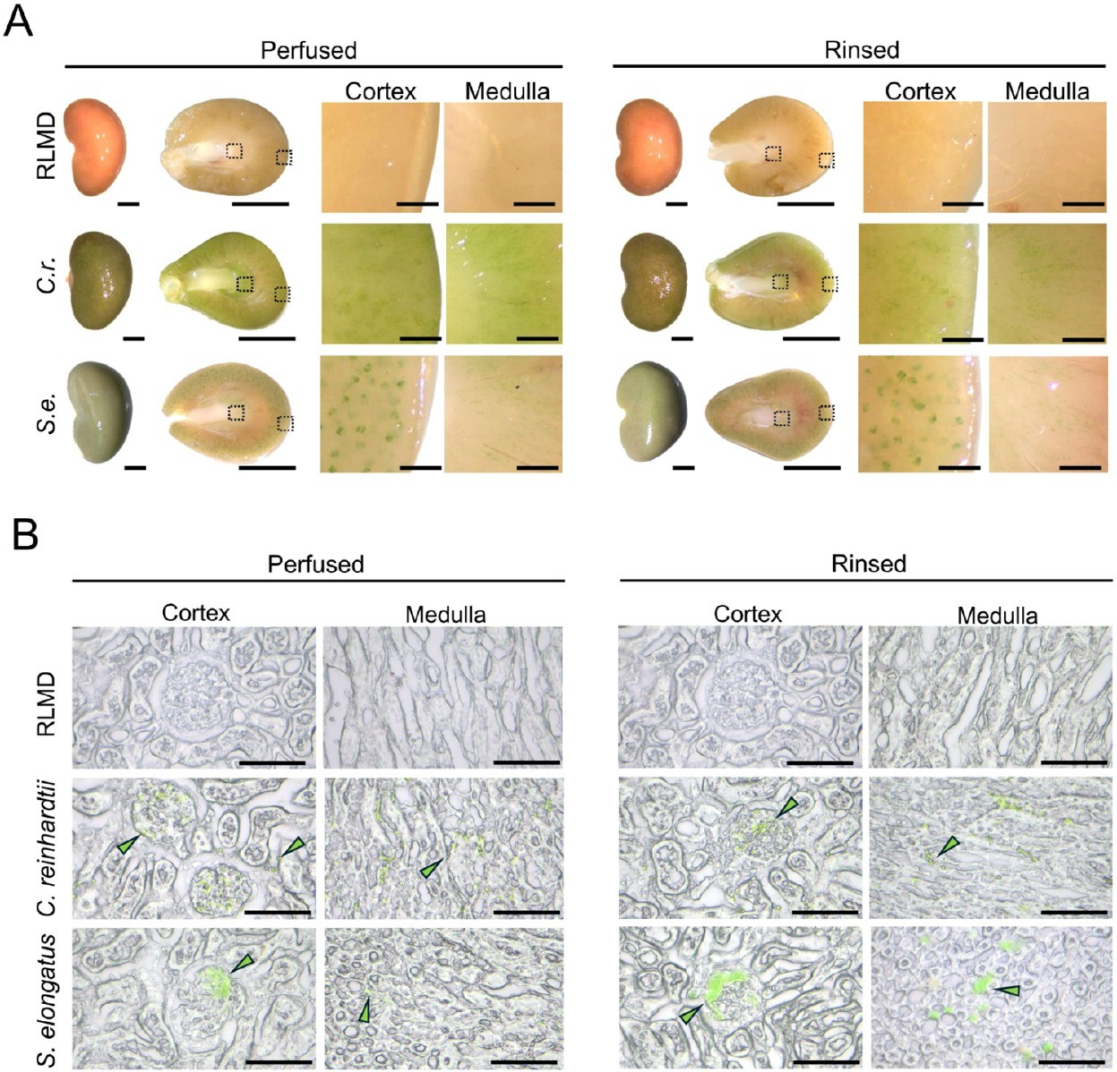
Distribution of microorganisms in perfused and rinsed kidneys. (A) From left to right: macroscopic view of whole kidneys, transversal slices, and magnified views of selected areas in the cortex and medulla of kidneys perfused with RLMD, (*C.r*.) or (*S.e*.), before (left) and after (right) rinsing. (B) Representative bright field images showing the distribution of photosynthetic microorganisms in cryosections of perfused and rinsed organs. In (B) green arrowheads show examples of photosynthetic microorganisms. In (A), the scale bars represent 5 mm for whole kidneys and transversal slices, and 0.5 mm for magnified views. In (B) scale bar represents 50 μm.

For better visualization and semiquantification, fluorescence microscopy analysis was performed in whole kidney slices and showed the persistence of both photosynthetic microorganisms in all vascular renal structures after rinsing with RLMD (Figure 7).

**Figure 7:**
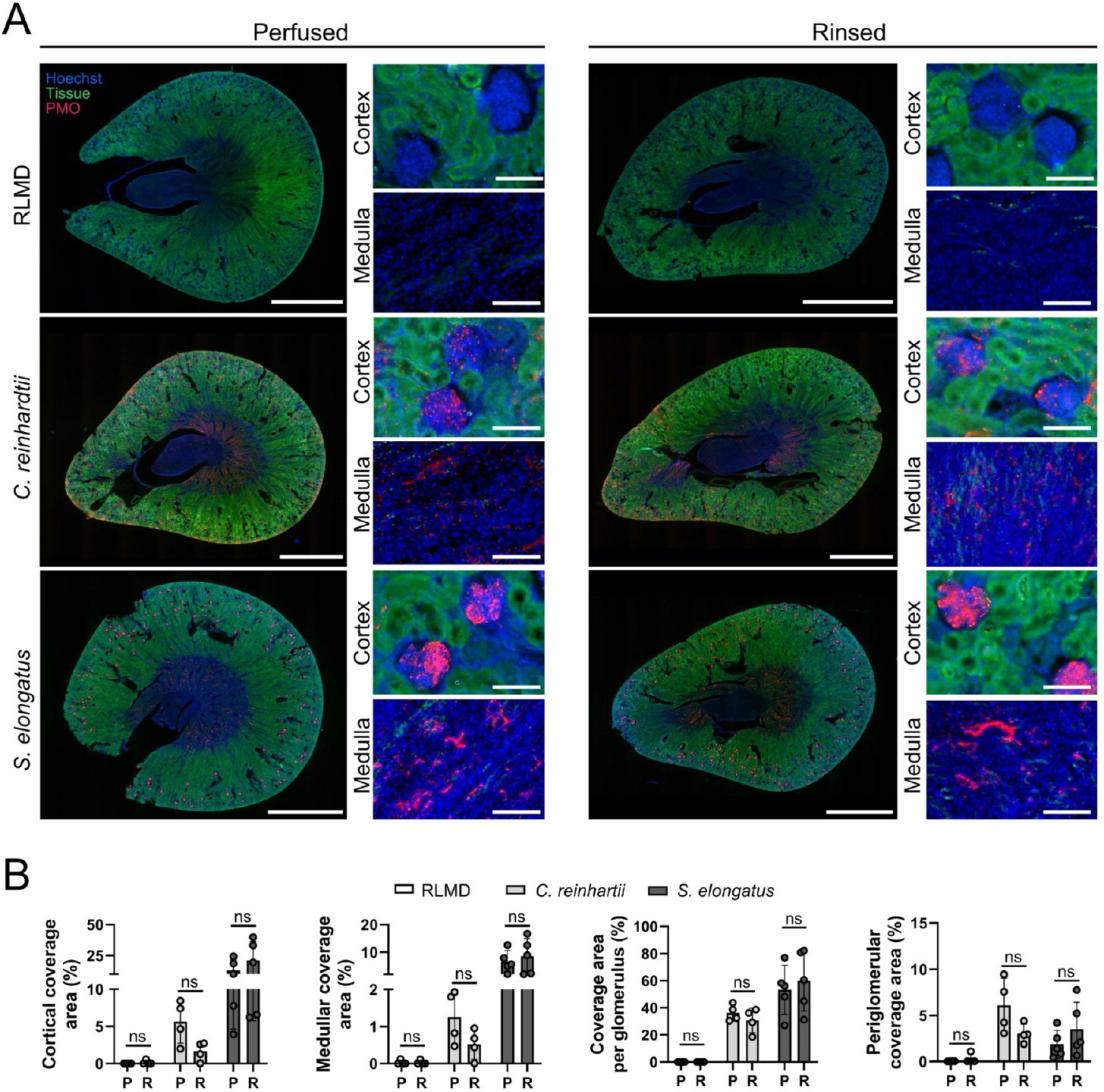
Distribution of perfused photosynthetic microorganisms in whole kidney slices. (A) Representative fluorescence images of kidney slices showing the distribution of photosynthetic microorganisms in cryosections of perfused and rinsed organs. From left to right, the quantifications in (B) correspond to photosynthetic microorganisms in the cortex, in the medulla, within each glomerulus, and in the cortical regions outside the glomeruli. Statistical analysis was performed using Two-way ANOVA followed by Sidak’s multiple comparisons test in (B, *N* ≥ 4). Data are presented as mean ± SD, and “ns” denotes nonsignificant differences. In (A), PMO stands for photosynthetic microorganisms. In (B), P and R represent the perfused and rinsed groups, respectively. Scale bars represent 3 mm for whole kidney slices and 100 μm for magnifications in (A).

### 3.4 *In Vitro* Interactions between Photosynthetic Microorganisms and Endothelial Cells

As no differences were observed between the perfused and rinsed kidneys, *in vitro* studies were conducted to explore the interactions between photosynthetic microorganisms and endothelial cells in more detail. A control solution (RLMD) or PSOP containing or were added to a monolayer of HUVECs and incubated for 1 h. After phalloidin staining (Figure 8A), monolayer integrity was quantified, and no significant differences were observed among groups (Figure 8B, upper panel). Furthermore, endothelial morphology was evaluated by examining actin coherence (Figure 8B, lower panel), revealing no differences among groups, with median coherence values of 0.22 (IQR: 0.13–0.39), 0.21 (IQR: 0.13–0.32), and 0.24 (IQR: 0.15–0.34) for HUVEC incubated with RLMD, microalgae, and cyanobacteria, respectively.

**Figure 8:**
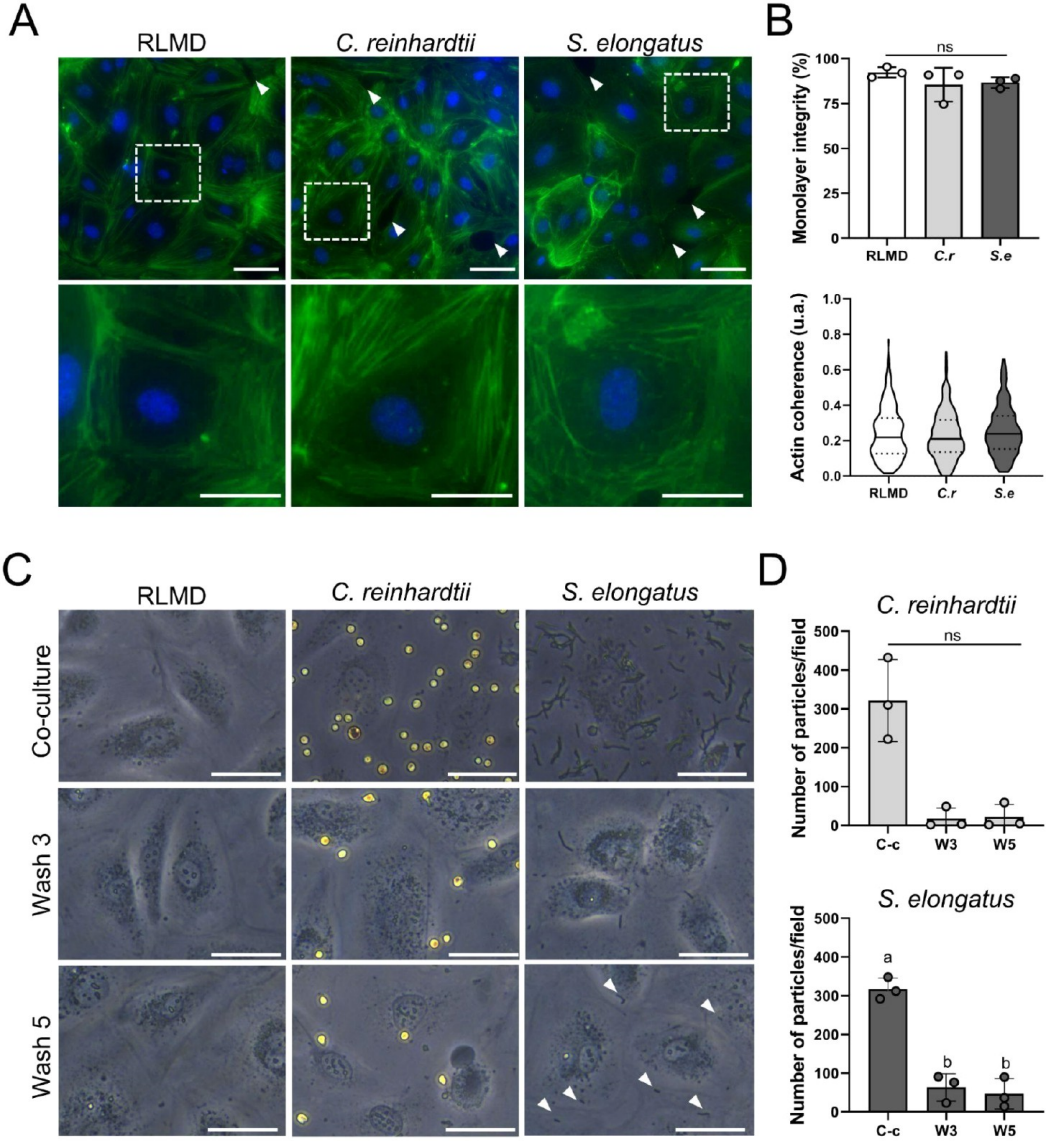
*In vitro* interaction between photosynthetic microorganisms and vascular endothelial cells. (A) Representative images of the actin cytoskeleton in HUVEC after coculture with RLMD, the microalgae , or the cyanobacteria . White arrowheads in the upper panel show monolayer discontinuities. In the lower panel, digital magnifications show a more detailed view of actin fibers. (B) Quantification of monolayer integrity and actin cytoskeleton coherence. In this violin plot, the continuous line shows the median, and dotted lines show the interquartile range. (C) Representative images of photosynthetic microorganisms in cocultures and adhered after washing steps 3 and 5. White arrowheads indicate undetached cyanobacteria remaining after the fifth wash. (D) Quantification of the number of microorganisms adhered to after each washing steps 3 and 5. Statistical analysis was performed using Shapiro-Wilk, Kruskal–Wallis test, followed by Dunn’s multiple comparisons test in (B, bottom) (*N* = 3). Shapiro-Wilk and Friedman test was conducted for data in (D, top) (*N* = 3). Shapiro-Wilk test and one-way ANOVA followed by Tukey’s multiple comparisons test in (D, bottom) (*N* = 3). Data are presented as mean ± SD. Statistical significance is indicated by different letters (*p* < 0.05), and “ns” denotes nonsignificant differences. In (B), *C.r*. denotes the microalga , and *S.e*. refers to the cyanobacterium . In (C), C-c, W3, and W5 indicate Coculture, wash 3, and wash 5, respectively. Scale bars correspond to 50 μm in (A, upper) and (C), and 25 μm in (A, lower).

To study whether a direct interaction between endothelial cells and photosynthetic microorganisms could partially explain their tissue persistence after rinsing *in vivo*, microalgae and cyanobacteria were washed off following coculture, and the remaining cells were visualized and quantified (Figure 8C). Notably, about 96% of microalgae and 79% of cyanobacteria were removed up to the second wash, with only a few cells remaining stably attached to the endothelial monolayer after five washes (Figure 8D). Interestingly, all clustered cyanobacteria seemed to be removed after the washing steps.

### 3.5 Quantification of Renal Tissue Damage Induced by PSOP Perfusion

Finally, to quantify and compare the potential damage induced by perfusion of both PSOP in the kidney, histological analyses were conducted using the EGT score. H&E staining (Figure 9A) revealed some alterations in renal structures, such as Bowman’s capsule thickening and edema. These characteristics were observed across all groups, including RLMD controls and samples perfused with both PSOP, before and after rinsing.

**Figure 9:**
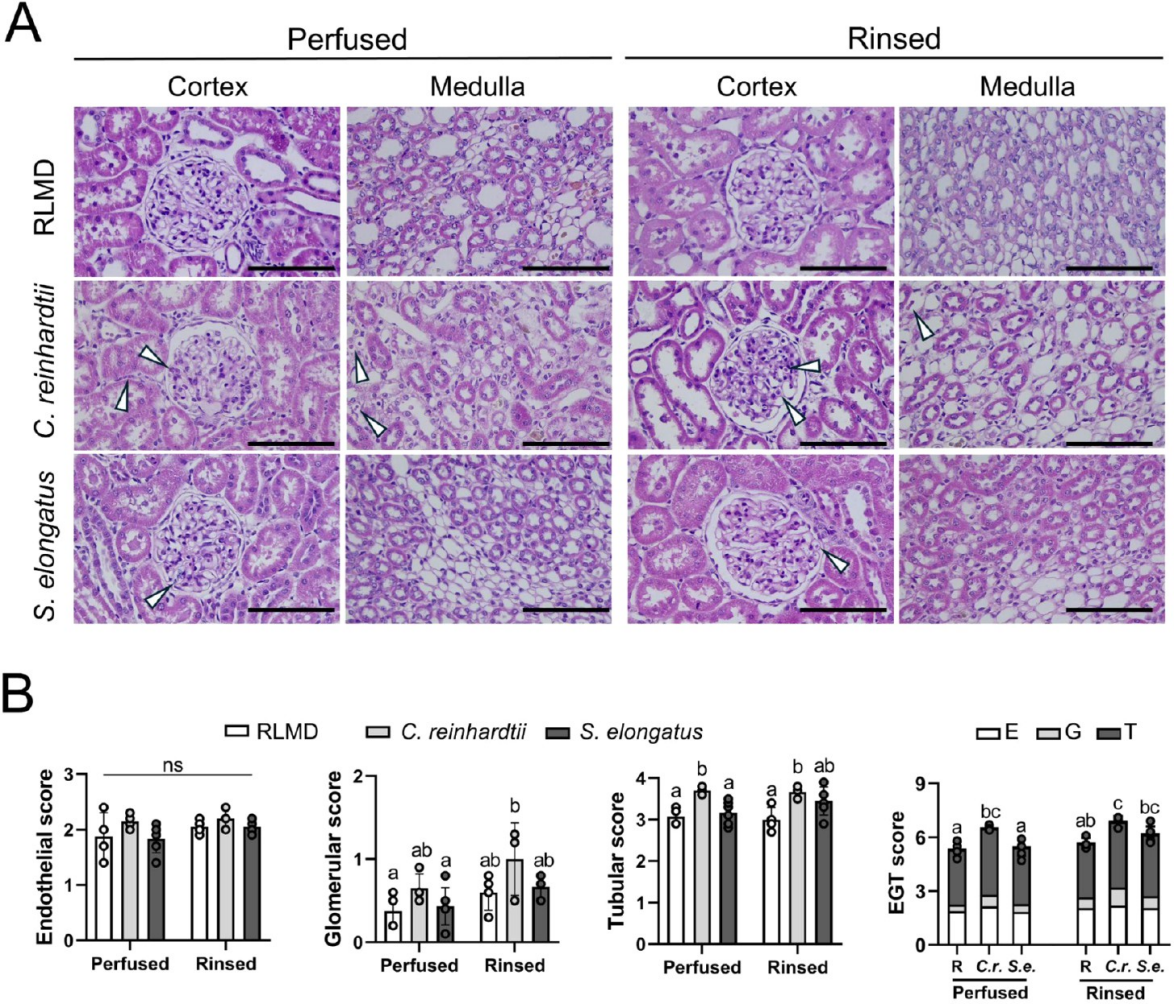
Histological analysis of tissue damage induced by perfusion with photosynthetic microorganisms. (A) Representative images of hematoxylin-eosin-stained sections of perfused rat kidneys before (left) and after (right) rinsing. White arrowheads in (A) indicate the presence of microorganisms in renal structures. (B) Quantification of tissue damage by Endothelial, Glomerular, Tubular, and overall EGT scores for the conditions depicted in (A). In (B, right), R represents the RLMD group, *C*.*r*. the microalgae , and *S.e*. the cyanobacteria . Statistical analysis was performed using two-way ANOVA followed by Tukey’s multiple comparison test (*N* ≥ 3). Data are presented as mean ± SD. Statistical significance is indicated by different letters (*p* < 0.05). Scale bar represents 20 μm in (A).

After quantification (Figure 9B), no significant differences were observed among groups in endothelial damage. Additionally, no significant differences were found in glomerular damage between the RLMD-perfused group and the cyanobacteria-perfused group. However, the group perfused with microalgae-based PSOP and subsequently rinsed showed significantly higher glomerular damage compared to the RLMD and cyanobacteria-perfused groups (before rinsing). Increased tubular damage was observed in the microalgae-perfused group before and after rinsing relative to the control group. For the total EGT score, the group perfused with microalgae showed a significantly higher score before rinsing; after rinsing, it remained higher only in comparison to the group perfused with RLMD.

## 4 Discussion

This study confirms the feasibility of delivering photosynthetic microorganisms into organs for intravascular photosynthesis, supporting previous studies with the microalga ,^13,18^ and extending the application to the cyanobacterium .

In this study, microalgae and cyanobacteria-based solutions were developed to produce equivalent oxygen levels, with a microorganism number ratio of 1:30, respectively. The density of was experimentally determined to sustain metabolically active organisms (zebrafish larvae), and the density of was adjusted to achieve a similar oxygen production rate.

Both microorganisms were able to maintain viability, proliferative ability, stable morphology, and oxygen production capacity in RLMD for at least 24 h. However, cyanobacteria-based PSOP exhibited lower oxygen consumption rates in the dark, which could be an advantage considering the technological challenges associated with illuminating solid organs to trigger intravascular photosynthesis.^19^ The limited penetration depth of visible light through biological tissues^25^ represents a significant hurdle for internal organ illumination. Strategies such as LED-based devices have been explored for confined implant spaces,^26^ and direct illumination was feasible in a small animal model with the open thoracic cavities.^27^ Another strategy tested in preclinical models involves the use of upconversion nanoparticles,^28–30^ which convert deeply penetrating near-infrared light into photosynthetically active visible wavelengths, enabling photosynthesis in deeper tissues. However, adapting such technologies for human-sized organs remains technically challenging. Currently, the absence of appropriate illumination devices complicates functional assays in whole organs, such as determining the duration of oxygen production within the vasculature, evaluating whether the generated oxygen is sufficient to prevent hypoxia, and assessing the viability and proliferation of photosynthetic microorganisms following illumination inside the organ.

In the context of organ preservation, the microalgae-based PSOP features were evaluated at 28 °C, whereas cyanobacteria-based PSOP was assessed at 37 °C. This was done to evaluate the performance of the microorganisms under their own optimal temperature.^31,32^ Moreover, this also aligns with subnormothermic and normothermic preservation strategies,^33^ showing the adaptability of microorganism-based preservation solutions to other existing innovative approaches for organ preservation.

To better understand the feasibility of PSOP in perfusion applications, their rheological properties were evaluated. In this study, viscosity measurements started at relatively high shear rates (>15 s^–1^), where viscosity appeared stable; nevertheless, the general profile suggests a pseudoplastic (shear-thinning) behavior, similar to that of blood.^34^ A marked shear thinning behavior has been reported at microorganism densities ten times higher than those employed here, such as 1 × 10^9^ cells/mL for microalgae-based PSOP.^13,18^

The viscosity of both PSOP, bearing approximately 1% microorganism volume fraction, did not differ from that of RLMD (vehicle), with an average value of around 2 mPa s, which is comparable to the viscosity of plasma or blood with a hematocrit ≤ 20%.^35–37^ *In vivo*, the Fahraeus-Lindqvist effect reduces hematocrit in small vessels (<300 μm in diameter), compensating for the shear-thinning behavior of blood and maintaining a relatively constant viscosity despite lower flow velocities in terminal arterioles and capillaries.^38^ Whether a similar reduction in volume fraction occurs in the vasculature when PSOP is perfused, as happens with erythrocytes when vessel diameter approaches cell size, remains unknown. However, such an effect is likely negligible at the low cell density used in this study (∼1% v/v). These considerations are particularly relevant to ensuring safe vascular perfusion during preservation procedures.

In this respect, the hemodynamic analysis of kidney perfusion showed no significant differences in vascular resistance among groups. However, the group perfused with the microalgae-based PSOP exhibited a tendency toward higher resistance and greater variability, starting from the perfusion and persisting through the rinsing step, suggesting some degree of vascular occlusion.

Unexpectedly, bright-field and fluorescence microscopy demonstrated the persistence of both photosynthetic microorganisms within renal structures after rinsing with RLMD, with each exhibiting distinct distribution patterns throughout the vascular network. Moreover, no significant quantitative differences in tissue persistence were observed between perfused and rinsed kidneys for either microorganism. The biconcave shape of erythrocytes and the lack of a nucleus and internal organelles allow deformability and facilitate their passage through narrow capillaries.^39^ In contrast, microalgae have a spherical shape and mean diameters of 5–6 μm, which could contribute to their retention within renal capillaries as narrow as 4.3 μm in diameter.^40,41^

Cyanobacteria, on the other hand, are significantly smaller than both microalgae and red blood cells, have a rod-like shape, and were therefore expected to pass through the capillary network without obstruction. However, only a portion was removed after the rinsing step, which could be partly explained by the unexpected formation of cyanobacterial clusters upon resuspension in RLMD. In fact, image analysis of cyanobacteria-based PSOP showed that approximately 25% of the counted particles had an area greater than 18 μm^2^, which was experimentally defined as the area of a single cyanobacterium in BG11 medium (data not shown). Subsequent *in vitro* interaction assays with endothelial cells demonstrated that most microorganisms, including cyanobacterial clusters, were effectively removed after washing, although a small fraction of single microorganisms remained adhered to the endothelial surface. Further studies are required to elucidate how individual and clustered cyanobacteria interact with endothelial cells *in vivo* and how this interaction could affect vascular perfusion dynamics.

Interestingly, analysis of venous perfusates showed that microorganisms maintain viability and shape after passing through the renal vasculature and revealed the presence of cyanobacterial clusters similar in size to those in the PSOP input solution, as well as microalgae with diameters larger than ∼5 μm. These findings challenge the assumption that particles with a size equal to or greater than the capillary diameter are retained within the microvasculature, suggesting that some degree of deformation of the cyanobacterial clusters or microalgae, the capillaries, or both occurred to allow their passage.

On the other hand, histological analysis of tissue damage revealed alterations consistent with ischemia-reperfusion injury in all groups of perfused kidneys, similar to those reported after approximately 45 min of ischemia.^24^ A comparable ischemic period likely occurred during this surgical protocol due to blood-flow disruption followed by the perfusion protocol, resulting in up to 40–50 min of ischemia for perfused and rinsed kidneys. Thus, similar tissue alterations were observed in RLMD control kidneys that underwent the same surgical and perfusion protocol without photosynthetic microorganisms. This further supports the notion that the observed damage is primarily attributable to the ischemic period, rather than the direct cytotoxic effects of the microorganism, thereby reinforcing its biocompatibility and highlighting the need to develop reliable and functional illumination devices to study the efficacy of vascular photosynthesis for tissue oxygenation. Importantly, no significant differences were found between the perfused control group and the perfused-and-rinsed control group in any of the evaluated components, despite the latter experiencing an additional 10 min of ischemia, suggesting that most ischemic injuries had already occurred prior to the rinsing stage.

Notably, no significant differences in endothelial or glomerular damage were found among the groups. In contrast, a greater tubular injury was evident in kidneys perfused with microalgae compared to other groups. The tubular compartment, which relies heavily on oxidative phosphorylation to sustain its high metabolic demand, demonstrated higher sensitivity to oxygen deprivation.^42^ Accordingly, while ischemia-induced damage was observed across all experimental groups, the greater tubular injury likely reflected the combination of surgical ischemia and the higher oxygen consumption rate of microalgae under dark conditions. Supporting this, subsequent *in vitro* assays, in which endothelial cells were exposed to RLMD or photosynthetic microorganisms, revealed only minor alterations in monolayer integrity, cell morphology, and cytoskeletal organization compared to RLMD control, suggesting no detrimental effect of the microorganisms on the cells and the preservation of their responsiveness to physiological stimuli such as blood flow direction and hormonal signals.^43,44^

Altogether, the results from hemodynamic, histological, and perfusate output analyses indicate that the size and shape of the microorganisms may not be critical factors in filling the vascular compartment and ensuring safe vascular perfusion during preservation procedures. However, further investigation is needed to optimize rinsing protocols, addressing the issue of microorganism clearance and whether residual microorganisms, although already described as innocuous and nonimmunogenic,^2,5–7^ may represent a significant impairment for organ recovery after implantation.

A drawback of this study is that the oxygenation capacity of cyanobacterial-based PSOP in perfused kidneys was not directly assessed. A previous study, which employed a microalgal-based PSOP, demonstrated that the oxygen provided by the microorganisms in the presence of light exceeded the metabolic requirements of the rat kidney slices,^13^ reinforcing the potential relevance of the approach of utilizing photosynthetic microorganisms in organ preservation. Nonetheless, further work is needed to optimize oxygen delivery, tailoring variables such as the employed microorganisms and preservation conditions to the specific needs for functional organ preservation. If higher oxygen levels are required, future strategies could include increasing cellular density within the organ’s vasculature, enhancing light penetration through optimized illumination systems, and genetically engineering strains with higher oxygen producing capacity or adapting microorganisms to function more effectively under mammalian physiological conditions. This could include variations in temperature, osmolality, and nutrient composition to ensure sustained viability and oxygen production throughout the preservation period. Complementary assessments, such as evaluating renal metabolic status, tissue integrity, and functionality after a clinically relevant preservation period under adequate light exposure, will provide more direct evidence of the efficacy of photosynthetic oxygen supply in preserving organ viability.

In summary, this study highlights the potential of using and in organ preservation strategies by demonstrating their viability, oxygen-producing capacity under physiological conditions, and feasibility for intravascular delivery into the renal vasculature. While these findings are encouraging, they also expose several technical and biological challenges that must be addressed before these systems can reach clinical relevance. In particular, although both strains produce oxygen, it remains uncertain whether the levels achieved under current conditions are sufficient to meet the metabolic demands of preserved organs. This highlights the need for further studies and the optimization of key parameters such as strain performance and effective light delivery during perfusion. Despite these challenges, the results presented here provide a valuable scientific foundation for developing PSOP-based approaches for intravascular photosynthesis in organ preservation and beyond.

## 5 Conclusions

This study confirms the feasibility of using microorganisms to develop photosynthetic solutions for organ perfusion for intravascular tissue oxygenation. By comparing two well-described model organisms, the microalga and the cyanobacteria , the results show that the chosen organism can be crucial in tailoring the PSOP for current *ex vivo* perfusion strategies. Interestingly, exhibited several advantages, including lower oxygen consumption in darkness, reduced variability in vascular resistance, and decreased tissue damage as compared with microalgae. Notably, although both organisms showed favorable hemodynamic profiles, they could only be partially removed under the tested experimental conditions, suggesting that vascular retention may be a complex issue influenced by factors beyond the microorganisms’ size and shape.

Overall, this work represents a significant step toward the development of biologically active perfusion solutions, offering novel opportunities for advancing tissue oxygenation. However, due to its novelty, further studies are required for the clinical translation of this approach, such as optimizing microorganism clearance and illumination, ensuring compatibility in circulation, and addressing proper *in situ* oxygen production.

## Supplementary Material





## Data Availability

All data associated with this study are in the paper and can be shared with approved outside collaborators under a materials transfer agreement; requests should be sent to J.T.E. jte@uc.cl.

## Notes

Hemodynamics of photosynthetic solutions for organ perfusion (PDF)
Surgery setting and perfusion of rat kidneys (AVI)

## ○

Department of Morphological Sciences, Faculty of Science, Universidad San Sebastian, General Lagos 1163, Valdivia 5090000, Chil
