# Supplementary material for: Photosynthetic Solutions for Organ Perfusion Based on Microalgae and Cyanobacteria Display Differential In Vitro and In Vivo Features for Intravascular Oxygenation
Source: ACS Appl Bio Mater. 2025 Aug 7;8(8):7433–48. doi: 10.1021/acsabm.5c01137 (PMC12365870; doi:10.1021/acsabm.5c01137)
Supplement: Supplementary file 1 [file mt5c01137_si_001.pdf]

Supporting Information

Photosynthetic Solutions for Organ Perfusion based on Microalgae and Cyanobacteria Display Differential *in Vitro* and *in Vivo* Features for Intravascular Oxygenation

Daniela Becerra[1], Valentina Vargas-Torres[1], Valentina Veloso-Giménez[1], Daniela Gallardo-Agüero[2], Miguel Miranda[1], Valentina Hernández-Pavez[1], Nicolás González-Quezada[1], Sebastián San Martín[2], Mauricio P. Boric[1], José Tomás Egaña[1]*.

[1] Institute for Biological and Medical Engineering, Pontificia Universidad Católica de Chile, Av. Vicuña Mackenna 4860, Santiago, 7820436, Chile.

[2] Center of Interdisciplinary Biomedical and Engineering Research for Health (MEDING), School of Medicine, Universidad de Valparaíso, Angamos 655, Viña del Mar, 2540064, Chile.

* Corresponding author: José Tomás Egaña (jte@uc.cl)

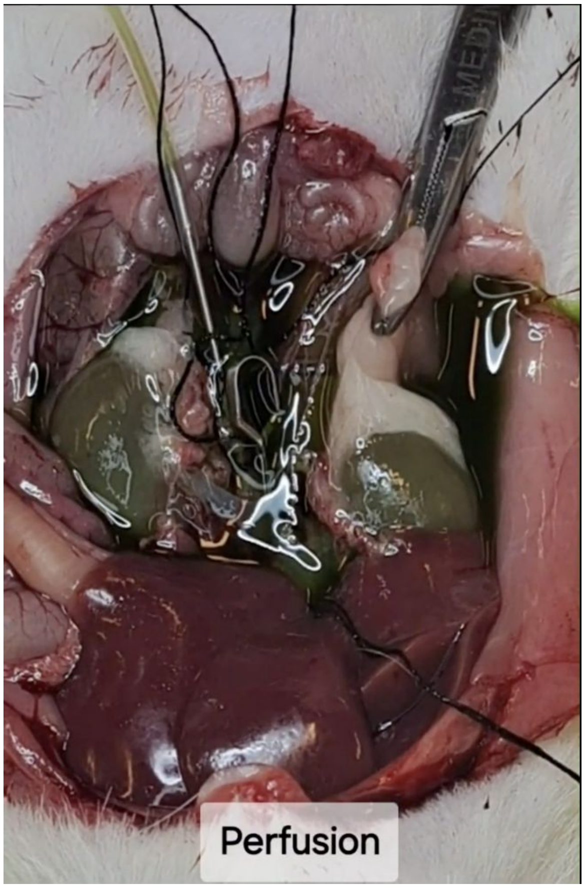

Video 1: Photosynthetic perfusion in a rat kidney model. The video shows the surgical process of perfusion with the Photosynthetic Solution for Organ Perfusion (PSOP), indicating the three separate stages: Flushing (removal of the blood), Perfusion (introduction of photosynthetic microorganisms into the renal vasculature), and Rinsing (removal of microorganisms from the vasculature).

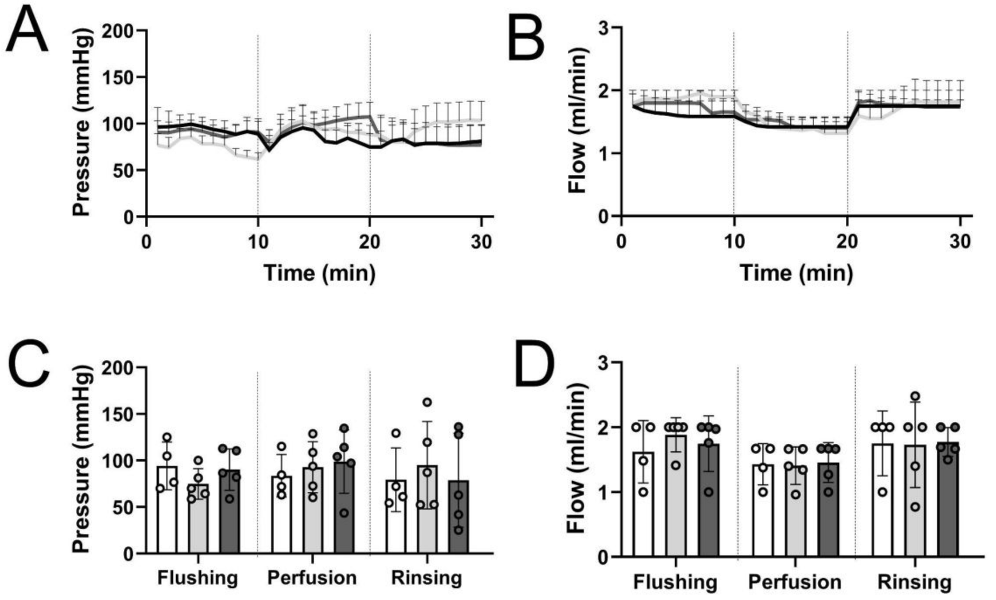

Figure S1: Hemodynamics of photosynthetic solutions for organ perfusion. (A) Time-course of renal pressure and (B) flow during the perfusion process. Bar graphs show mean (C) pressure and (D) flow in each stage. Each dot represents an animal. Statistical analysis was performed using two-way ANOVA followed by Tukey's multiple comparison test in (B) ($n \geq 4$). No significant differences were observed between groups in (A-D) ($p < 0.05$).
